# Drug-induced lung disease: a narrative review

**DOI:** 10.36416/1806-3756/e20240110

**Published:** 2024-09-16

**Authors:** Guilherme das Posses Bridi, Eduardo Kaiser Ururahy Nunes Fonseca, Ronaldo Adib Kairalla, Alexandre Franco Amaral, Bruno Guedes Baldi

**Affiliations:** 1. Divisao de Pneumologia, Instituto do Coracao (InCor), Hospital das Clinicas, Faculdade de Medicina, Universidade de Sao Paulo - HCFMUSP - São Paulo (SP) Brasil.; 2. Núcleo de Pulmão, AC Camargo Cancer Center, São Paulo, Brasil.; 3. Instituto de Radiologia, Hospital das Clínicas, Faculdade de Medicina, Universidade de São Paulo - HCFMUSP - São Paulo, SP, Brasil.; 4. Grupo de Radiologia Cardiotorácica, Hospital Israelita Albert Einstein, São Paulo (SP) Brasil.; 5. Núcleo de Tórax, Hospital Sírio-Libanês, São Paulo, Brasil.; 6. Hospital do Coração, São Paulo (SP), Brasil.

**Keywords:** Lung diseases, interstitial/chemically induced, Lung diseases, interstitial/diagnostic imaging, Immunotherapy/adverse effects

## Abstract

Drug-induced lung disease (DILD) encompasses a broad, highly heterogeneous group of conditions that may occur as a result of exposure to numerous agents, such as antineoplastic drugs, conventional or biological disease-modifying antirheumatic drugs, antiarrhythmics, and antibiotics. Between 3% and 5% of prevalent cases of interstitial lung diseases are reported as DILDs. The pathogenesis of lung injury in DILD is variable, multifactorial, and often unknown. Acute presentation is the most common, can occur from days to months after the start of treatment, and ranges from asymptomatic to acute respiratory failure. The CT patterns are varied and include ground-glass opacities, organizing pneumonia, and diffuse alveolar damage. Notably, there are no clinical manifestations or CT patterns specific to DILD, which makes the diagnosis quite challenging and necessitates a high index of suspicion, as well as the exclusion of alternative causes such as infection, cardiac-related pulmonary edema, exacerbation of a preexisting ILD, and neoplastic lung involvement. Discontinuation of the offending medication constitutes the cornerstone of treatment, and corticosteroid treatment is usually necessary after the onset of clinical manifestations. The prognosis varies widely, with high mortality rates in severe cases. A history of medications related to pulmonary toxicity in patients with new-onset respiratory symptoms should prompt consideration of DILD as a potential underlying cause.

Drug-induced lung disease (DILD) constitutes a significant, heterogeneous group of adverse drug reactions that occur after exposure to various medications. To date, more than 500 drugs have been associated with the emergence of DILD, a number that rises every year, particularly with the increasing use of antineoplastic drugs, immune checkpoint inhibitors (ICIs), antiarrhythmics, antibiotics, and disease-modifying antirheumatic drugs (DMARDs).^1^ ^,^ ^2^

Making a diagnosis of DILD can be challenging if there are preexisting lung conditions such as those induced by radiotherapy, as well as COPD, inflammatory lung disease, and interstitial lung disease (ILD). The clinical and radiological manifestations of DILD are nonspecific, often exhibit an association with the initiation of treatment of a given drug, typically emerging within the first three months of treatment, and may range from mild to severe and life-threatening.^2^ ^,^ ^3^ The present article aims to comprehensively review the spectrum of drug-induced diseases of the lung parenchyma, as well as presenting updated approaches to their diagnosis and management.

## EPIDEMIOLOGY

Estimating the incidence of DILD is challenging, because it can vary depending on the population demographics and the treatments available within regional healthcare systems. In studies of patients with non-small cell lung cancer (NSCLC), the overall incidence of DILD across all grades ranges from 1.4% to 5.8%.^3^ ^,^ ^4^ Among patients with autoimmune diseases, especially rheumatoid arthritis (RA), the prevalence of DILD is 0.3-11.0% in those treated with methotrexate and 0.5-3.0% in those treated with anti-TNF agents.^5^ Severe (grade 5) pneumonitis occurs in 2-9% of patients, with higher incidences in individuals who have preexisting lung conditions and are using more than one drug simultaneously, often resulting in mortality rates as high as 36%,^2^ ^,^ ^6^ as shown in Chart 1.

**Chart 1:** Grades of pneumonitis severity.

Grade	Description
1 (mild)	Asymptomatic, radiographic findings only
2 (moderate)	Symptomatic, not interfering with activities of daily living
3 (severe)	Severe symptoms, involving > 50% of lung, and requiring hospitalization
4 (life-threatening)	Life-threatening respiratory involvement, ventilator support required
5 (fatal)	Fatal outcome attributed to drug-induced pulmonary toxicity

Adapted from Delaunay et al.^6^

In recent cohorts of ILD patients, between 3% and 5% of prevalent cases are reported as drug-induced, translating to an annual incidence of DILD ranging from 4.1 to 12.4 cases per million population. This incidence may be underestimated given the challenges in diagnostic confirmation as well as the emergence of new drugs and treatment combinations.^2^

## PATHOGENESIS

The mechanisms of lung injury in DILD are variable and usually multifactorial, depending on the particular drug involved. Key mechanisms include cytotoxic effects on alveolar capillary endothelial cells, immune-mediated lung injury, pulmonary drug deposition, oxidative stress, and immune system dysregulation. Unfortunately, the precise pathogenesis is unknown for many drugs.^2^

Although DILD generally affects the lung parenchyma, it can also involve the airways, giving rise to a variety of clinical and histological patterns, including hypersensitivity reaction, pulmonary fibrosis, bronchospasm, pneumonitis, and noncardiogenic pulmonary edema.^7^ The interaction between individual factors, such as genetics and previous or current exposures, may predispose individuals to pulmonary toxicity. In Japanese patients, for instance, the presence of HLA-DRB1*04:05 and HLA-B*15:01 alleles has been linked to pulmonary toxicity.^2^ ^,^ ^8^ In contrast, the presence of the HLA-A*3101 allele has been associated with drug-induced hypersensitivity reactions in individuals of European descent.^9^

## RISK FACTORS

The likelihood of pulmonary adverse events is influenced by factors such as the agents/dosages administered, exposure duration, and intrinsic (patient) risk factors.^10^ Therefore, although the development of DILD is often unpredictable, some individual factors are associated with a higher risk of pulmonary toxicity (Chart 2). For instance, cumulative dose, renal dysfunction, advanced age, and stage IV disease at presentation confer an increased risk of pulmonary toxicity in patients treated with bleomycin.^11^ Similarly, genetic factors, preexisting ILD, male sex, smoking, and poor performance status are more associated with adverse events in patients treated with EGFR tyrosine kinase inhibitors (TKIs) or chemotherapeutic agents.^12^ ^,^ ^13^

**Chart 2:** Main risk factors for drug-induced lung disease.

Advanced age (> 40 years of age)
Renal dysfunction (GFR < 80 mL/min)
Dose-dependent toxicity
Genetic predisposition (familial pulmonary fibrosis, Japanese descent)
Previous radiotherapy
Preexisting ILD
Exposure to high FiO_2_
Concurrent administration of pneumotoxic drugs
Poor performance status or advanced lung cancer
Smoking
COPD

## CLINICAL, FUNCTIONAL, AND RADIOLOGICAL MANIFESTATIONS

Symptoms of DILD are nonspecific and can manifest within days or years after exposure to the offending drug(s). Acute pneumonitis typically results in shortness of breath, cough, fever, and peripheral eosinophilia, with some patients evolving to acute respiratory failure.

In cases of subacute or chronic disease, particularly those with prolonged exposure, signs of pulmonary fibrosis are common and the main symptoms are worsening dyspnea and reduced exercise capacity. Physical examination may reveal fine or “velcro-like” crackles, whereas digital clubbing is uncommon. In advanced fibrotic disease, there can be signs of pulmonary hypertension (PH) and right ventricular dysfunction.^2^ ^,^ ^5^ In the majority of DILD cases, pulmonary function tests reveal a pattern of restrictive abnormality, although an obstructive pattern may also be seen.^14^

Radiological patterns reflect the generally inflammatory nature of DILD. The most common disease patterns include diffuse alveolar damage (DAD), organizing pneumonia (OP), nonspecific interstitial pneumonia (NSIP), eosinophilic pneumonia, and sarcoid-like reaction.^2^ Details regarding the manifestations of the main drugs that may determine DILD are provided below (Chart 3). 

**Chart 3:** Main patterns of pulmonary toxicity and the potential treatments involved.

Acute or subacute ILD	TNF-α antagonists, ICIs, conjugated antibodies, chemotherapy, TKIs, amiodarone, nitrofurantoin, mTOR inhibitors, rituximab, abatacept, methotrexate, leflunomide, azathioprine
Organizing pneumonia	Amiodarone, antineoplastics (including ICIs), TNF-α antagonists, rituximab, sulfasalazine, mTOR inhibitors, minocycline, anticonvulsants, radiation therapy
Pulmonary fibrosis	Chemotherapy (bleomycin, cyclophosphamide, carmustine, busulfan, gemcitabine, amiodarone, nitrofurantoin, and methotrexate)
Eosinophilic pneumonia	Antibiotics (minocycline and nitrofurantoin), anticonvulsant, amiodarone, antidepressants, aspirin, chloroquine, mesalazine, nitrofurantoin, tryptophan, dupilumab, imatinib
Sarcoid-like reaction	BCG therapy, TNF-α antagonists, interferon, breast implant, HAART, pirfenidone, ustekinumab, ICIs, vemurafenib
Noncardiogenic pulmonary edema	Nitrofurantoin, TMP-SMX, all-trans-retinoic acid, aspirin, chemotherapy, cocaine, heroin, hydrochlorothiazide, i.v. epoprostenol, opioids
Pleuroparenchymal fibroelastosis	Cyclophosphamide, alkylating agents (including carmustine), daptomycin, statins
Hypersensitivity pneumonitis	Methotrexate, isocyanates, cannabis, irinotecan
Airway disease	ICIs (durvalumab, pembrolizumab), rituximab, cocaine, vaping, chlorine gas, penicillamine
Alveolar proteinosis	Chemotherapy, cyclosporine, mTOR inhibitors, imatinib, leflunomide

ICIs: immune checkpoint inhibitors; TKIs: tyrosine kinase inhibitors; mTOR: mechanistic target of rapamycin; BCG: bacillus Calmette-Guérin; HAART: highly active antiretroviral therapy; TMP-SMX: trimethoprim-sulfamethoxazole; and i.v.: intravenous. Adapted from Spagnolo et al.^2^

## BIOLOGIC AGENTS

In clinical practice, the utilization of biologic agents for the treatment of autoimmune diseases has expanded significantly in recent years. That has resulted in an increase in the occurrence of pulmonary toxicity. Anti-TNF agents have been mostly associated with DILD or exacerbation of a preexisting ILD, as well as being frequently associated with infectious and noninfectious granulomatous lung disease, DAD, and, less often, pulmonary fibrosis and OP.^15^ ^,^ ^16^ In addition, other diseases, such as lupus, vasculitis, autoimmune hepatitis, sarcoidosis, uveitis, and demyelinating neurologic diseases, may be triggered by biologic agents.^17^ Positivity for antinuclear antibodies (ANA) may also occur during or after the use of such drugs, with the majority of patients remaining asymptomatic.^18^

### Infliximab

Infliximab, a chimeric monoclonal antibody, inhibits TNF-α and has been approved for the treatment of inflammatory bowel diseases, psoriasis, psoriatic arthritis, ankylosing spondylitis, RA, and severe sarcoidosis.^19^ The incidence of anti-TNF-induced ILD ranges from 0.5% to 3.0%, and pulmonary radiological features attributed to the use of infliximab include aseptic granulomatous pulmonary nodules, interstitial lung infiltrates, eosinophilic pneumonia, and acute respiratory distress syndrome (ARDS).^1^ ^,^ ^20^ ^,^ ^21^

### Adalimumab

**Figure 1:**
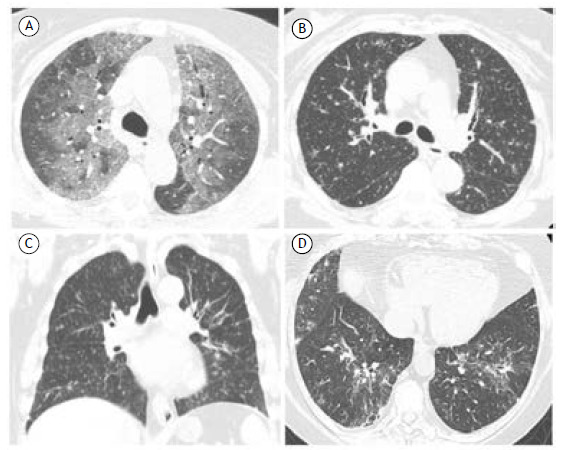
In A, axial CT scan showing pulmonary ground-glass opacities, predominantly in the upper lobes, in a 62-year-old female patient with RA after receiving the second dose of adalimumab. In B and C, CT scans showing diffuse, predominantly perilymphatic, micronodules in a patient with RA and sarcoid-like reaction associated with the use of etanercept. In D, axial reconstruction of a CT scan showing ground-glass opacities and organizing pneumonia patterns in the lower lobes of a patient with systemic lupus erythematosus after rituximab use.

Adalimumab-induced ILD is rarely described. Predictors of a poor prognosis for such complications include age > 65 years, late onset of symptoms, concomitant use of other immunosuppressants, especially methotrexate, and a previous diagnosis of ILD. The mean time to symptom onset is 26 weeks, and the disease may evolve to acute respiratory failure. In the largest sample of patients using adalimumab, imaging modalities like HRCT revealed ground-glass opacities (GGO, Figure 1A) in 83%, honeycombing in 22%, and reticulonodular, sometimes diffuse, opacities in 38%.^15^ ^,^ ^17^

### Etanercept

Etanercept was the first specific anti-cytokine therapy approved for the treatment of RA. It can also be used to treat psoriatic arthritis and ankylosing spondylitis. Among patients with RA, sarcoidosis-like disease is more common in those receiving etanercept. On CT, the features are similar to the typical findings of sarcoidosis, including perilymphatic micronodules and lymphadenopathy.^22^ In addition, in etanercept-treated patients with RA (Figures 1B and 1C), it has been shown that 11.0-36.3% of such patients develop ANA positivity and 5.0-15.0% develop positivity for anti-double-stranded DNA antibodies.^(16-18, 23)^

### Rituximab

The anti-CD20 antibody rituximab is widely used for the treatment of malignant lymphoma and various autoimmune disorders, including RA. Acute lung injury with bilateral opacities has been reported to occur at any time during treatment, mostly in patients with neoplastic/hematologic disorders, and may be fatal. Although the mechanism of rituximab-induced lung damage is unknown, it has been suggested that it is related to lysis of neoplastic cells and release of cytokines.^2^ ^,^ ^24^ Most cases of rituximab-induced ILD occur on average 3 months after the first infusion but may occur within the first 24 h after the first injection. Organizing pneumonia and alveolar hemorrhage, as shown in Figure 1D, have also been described.^2^

### Abatacept

Abatacept is a biologic DMARD characterized by a fusion protein comprising CTLA-4. It appears to be an effective treatment for patients with RA-associated ILD and can be used in patients with a history of previous pulmonary infection. Although few cases of pulmonary toxicity have been attributed to abatacept compared with other therapeutic agents, such cases have shown a rapid progression to respiratory failure.^25^

### Tocilizumab

Tocilizumab is an anti-IL-6 receptor antibody indicated for the treatment of patients with RA, systemic or polyarticular juvenile idiopathic arthritis, or giant cell arteritis. It has also been shown to have the effect of slowing the FVC decline in patients with ILD associated with systemic sclerosis.^26^ Pulmonary toxicity is uncommon, with the primary complications being the occurrence of pneumonia and opportunistic infections. However, pneumonitis, OP, and sarcoid-like reactions have been described.^27^ ^,^ ^28^

## ANTINEOPLASTIC THERAPY

Pulmonary toxicity has been reported to occur in 10-20% of all patients treated with antineoplastic drugs, typically within weeks or a few months after treatment initiation. Identifying specific causative agents is challenging, especially due to the combination of drugs and radiotherapy, which increases the risk of pulmonary toxicity in patients with lung cancer. Radiological abnormalities include patchy or diffuse GGO, consolidation, centrilobular nodules, interlobular septal thickening, and reticular changes. The prognosis is uncertain and typically worse in patients who have a high burden of previous lung disease.^2^

### Platinum analogues

Platinum-based chemotherapy is frequently used in the treatment of various cancers, such as colorectal, lung, and genitourinary cancer. Various cases of oxaliplatin-induced interstitial pneumonia have been reported over the past few decades.^29^ Shimura et al.^30^ reported that smoking, pulmonary metastasis, and the presence of a preexisting lung disease were linked to a higher risk of developing platinum-induced pulmonary toxicity. Lung lesions typically develop after 5 or 6 cycles of treatment, often presenting as interstitial pneumonia and GGO. The use of carboplatin and cisplatin can also lead to ARDS and eosinophilic pneumonia.^1^

### Taxanes

**Figure 2:**
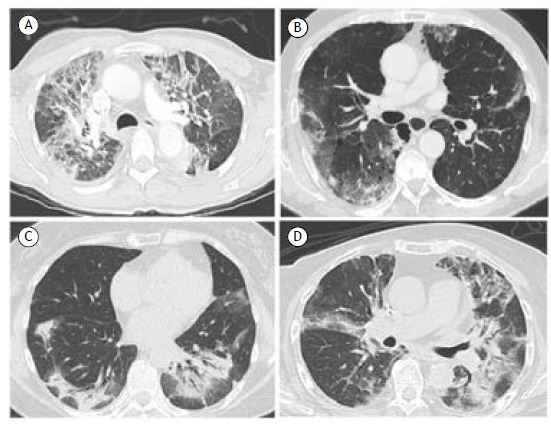
In A, axial CT scan showing pulmonary ground-glass opacities and organizing pneumonia, predominantly in the upper lobes, in a female patient with squamous non-small cell lung cancer using docetaxel. In B, CT showing ground-glass opacities overlapping with areas of emphysema, particularly in the right lower lobe, in an elderly patient with lung cancer using pembrolizumab. In C, CT scan showing bilateral areas of consolidation in the lower lobes, consistent with organizing pneumonia, in a 62-year-old female patient with melanoma under treatment with nivolumab plus ipilimumab. In D, axial CT scan shows ground-glass opacities and consolidations suggestive of organizing pneumonia in middle and upper lobes in a patient with breast cancer treated with trastuzumab-deruxtecan.

Taxanes, which include paclitaxel and docetaxel, are a class of mitotic inhibitors. The combination of platinum and taxane therapy is widely employed in the treatment of lung cancer.^31^ Retrospective studies have reported an overall incidence of pulmonary toxicity as high as 4.6% in NSCLC patients receiving docetaxel therapy, with the median onset of symptoms being 18 days after the last administration.^32^ In patients with preexisting pulmonary fibrosis treated with a taxane (Figure 2A), the reported rate of grade 3 or higher pneumonitis is 27%.^33^

### Antifolates

Pemetrexed is a multitargeted antifolate used in the treatment of malignant pleural mesothelioma and NSCLC. In Japanese cohorts, pemetrexed toxicity was reported to occur in approximately 3.6-4.0% of cases, with the main patterns being GGO and acute interstitial pneumonia. Methotrexate, a similar antifolate, is known to induce steroid-responsive ILD in rheumatic patients (see below), and similar occurrences have been reported in cases of pemetrexed toxicity.^34^

### EGFR-TKIs

Pneumonitis is noted as a feature of class-effect toxicity of EGFR-TKIs, including gefitinib, erlotinib, afatinib, cetuximab, and osimertinib. The overall incidence of all-grade DILD in patients treated with an EGFR-TKI reportedly ranges from 0% to 5.3%, with a low risk of recurrence in patients retreated after the initial exposure.^12^ ^,^ ^35^ Risk factors include a significant smoking history, advanced age, preexisting ILD, poor performance status, recent NSCLC diagnosis, and involvement of > 50% of lung areas.^12^ The HRCT patterns are similar to those seen with cytotoxic agents, which most commonly manifest as diffuse GGO, OP, or a fibrotic pattern.^1^ ^,^ ^2^

### Anaplastic lymphoma kinase-TKIs

The incidence of pulmonary toxicity in patients with advanced NSCLC using the anaplastic lymphoma kinase-TKIs crizotinib and alectinib is estimated to be approximately 1.8% and 2.6%, respectively. In Japanese patients, the incidence may be higher (up to 5.77%), especially in smokers and the elderly, and symptoms begin approximately 4-8 weeks after treatment initiation. The most commonly reported patterns are OP and DAD.^36^

### Antineoplastic antibiotics

Bleomycin is a polypeptide antineoplastic antibiotic that is commonly used in the treatment of lymphoma and germ-cell tumors. Bleomycin-induced pulmonary toxicity has been recognized since the early clinical trials in the 1960s, and it is one of the drugs with the greatest potential for pulmonary toxicity. Such toxicity is predominantly fibrotic and appears to be immune-mediated by macrophages and lymphocytes secreting TNF, together with hypersensitivity reactions, the production of reactive oxygen radicals, and cellular toxicity. Major risk factors for bleomycin-induced pulmonary toxicity include age > 40 years, chronic kidney disease, cumulative dose > 300,000 IU, and stage IV disease at presentation. Several distinct pulmonary syndromes are associated with bleomycin use, including OP, eosinophilic pneumonia, and, most commonly, a diffuse GGO pattern related to DAD,^11^ ^,^ ^37^ ^,^ ^38^ as depicted in Figure 4D.

Doxorubicin is an anthracycline antibiotic used in the treatment of solid tumors such as breast cancer, as well as leukemia and lymphoma. Treatment with doxorubicin is limited by its cardiac toxicity, dose dependent congestive heart failure, and cardiomyopathy. Rare cases of pneumonitis and progression to fibrotic ILD have been described.^39^

### Antineoplastic drugs

Capecitabine is an oral prodrug of 5-fluorouracil. The clinical efficacy of capecitabine has been demonstrated in the treatment of gastric, colorectal, and breast cancer. Only a few adverse pulmonary events have attributed to the drug, most commonly a sarcoid-like reaction, mediastinal/hilar lymphadenopathy, and airway involvement.^40^

Irinotecan is a chemotherapeutic agent that is widely used for the treatment of colorectal, gastric, lung, and breast cancer. The incidence of DILD after irinotecan use is low (approximately 1%). The most common patterns are DAD, OP, and hypersensitivity pneumonitis.^41^

Fludarabine is a nucleoside analog that is widely used in the treatment of low-grade lymphoproliferative malignancies including chronic lymphocytic leukemia and low-grade non-Hodgkin lymphoma. In such cases, the incidence of pulmonary toxicity is approximately 8.6%. Acute interstitial pneumonia, OP, eosinophilic pneumonia, and pulmonary nodules are the most common abnormalities described.^42^ ^,^ ^43^

Chlorambucil is an alkylating agent also used for the treatment of indolent lymphoproliferative disorders such as chronic lymphocytic leukemia. Pulmonary toxicity has been reported as a dose-independent adverse effect occurring during or after treatment with chlorambucil. Events may range from acute interstitial pneumonitis, OP, and bronchiolar disorders to signs of pulmonary fibrosis after prolonged exposure.^44^

### Proteasome inhibitors

Bortezomib is a proteasome inhibitor, currently used as the primary treatment for multiple myeloma (MM) and lymphoma worldwide. Severe pulmonary complications have been reported, the most common findings being acute pneumonitis, OP, and ARDS. The incidence rate is approximately 4.5%, with a mortality of 0.5%, mostly in the Japanese population.^1^ ^,^ ^45^

### Immunomodulators

The immunomodulatory drugs thalidomide and lenalidomide are also indicated for the treatment of MM. Lenalidomide is a less toxic, more powerful immunomodulator than is thalidomide and is typically used in combination with dexamethasone in refractory cases. The mechanism of lung injury remains unclear; acute pneumonitis, OP, and eosinophilic pneumonia are the most common lesions described.^1^ ^,^ ^46^

### Alkylating agents

Busulfan is an alkylating agent that was initially used in the treatment of chronic myelogenous leukemia but is currently used exclusively as a component of different conditioning regimens preceding allogeneic hematopoietic stem cell transplantation. Pulmonary toxicity is estimated to occur in up to 8% of patients using busulfan. Risk factors for DILD after busulfan use include a cumulative dose of more than 500 mg, concomitant administration of additional drugs associated with pulmonary toxicity, and lung irradiation. The most common patterns of pulmonary toxicity are DAD and OP, although pulmonary alveolar proteinosis may also occur.^1^ ^,^ ^47^

Carmustine is a nitrosourea that is widely used to treat malignant brain tumors, Hodgkin/non-Hodgkin lymphoma, and MM. Pulmonary toxicity can present as DAD, ARDS, radiation recall pneumonitis with acute symptoms, and pleuroparenchymal fibroelastosis (PPFE).^48^

### Pyrimidine analogues

Gemcitabine is a pyrimidine analog that is widely used to treat solid tumors such as breast, colon, ovarian, and pancreatic cancer, as well as NSCLC. The frequency of gemcitabine-related pulmonary toxicity is estimated to range from 2.7% to 24.0%, with few severe cases. The most common patterns are NSIP, hypersensitivity pneumonitis-like lesions, alveolar hemorrhage and radiation recall pneumonitis. The combination of gemcitabine and carboplatin also induces a significant decrease in diffusion capacity.^49^ ^,^ ^50^ However, the mechanisms of pulmonary toxicity remain unclear.

## IMMUNOTHERAPY

Immunotherapy revolutionized the treatment of cancer with the development of ICIs, which have a broad range of indications, including lung cancer, melanoma, bladder cancer, and head and neck tumors.^2^ However, ICIs can also induce specific hyperactivation of the immune response, leading to systemic tissue damage. Immune-related adverse events, such as rash, colitis, hepatitis, myocarditis, endocrine disorders, and pneumonitis, are commonly reported. The incidence of pneumonitis varies from 3% to 6%, including 1-2% of grade 3-4 adverse events.^2^ ^,^ ^51^ Immunotherapies may initially provoke an increase in tumor size or the development of new lesions as pseudoprogression. Immunotherapy-mediated pseudoprogression is defined as a ≥ 25% increase in tumor burden that is not seen on repeated imaging performed ≥ 4 weeks after the initial study.^52^

### PD-1 inhibitors

Pembrolizumab is an antibody against programmed cell death 1 (PD-1) that increases anti-tumor T-cell responses by blocking the interaction between PD-1 on T cells and its ligand (PD-L1) on cancer cells. The main risk factors for pembrolizumab-induced pulmonary toxicity include age > 70 years, prior thoracic radiation, previous lung disease (COPD, asthma, or ILD), combination therapy (chemotherapeutic drugs or ICI followed by osimertinib), smoking status, and histological type of NSCLC (squamous NSCLC).^53^ The most common patterns are diffuse GGO and OP, followed by fibrotic NSIP and centrilobular ground-glass nodule patterns (Figure 2B).^37^

Nivolumab, another antibody that targets PD-1, is currently used for the treatment of patients with malignant melanoma, NSCLC, renal cell carcinoma, Hodgkin lymphoma, and head and neck cancer. The onset of pulmonary toxicity varies from within a few days to more than a year after initiation, with a median of 3 months after use of the drug.^2^ Studies have shown that the prevalence of pneumonitis caused by nivolumab (Figure 2C) is low (2.9%), although it increases significantly (to 11.8%) when nivolumab is combined with another ICI.^54^ Up to 10% of patients with advanced NSCLC have a preexisting ILD, and nivolumab seems to be safe in this population.^55^

### PD-L1 inhibitors

Atezolizumab is a humanized IgG1 monoclonal antibody against PD-L1, used in tumors such as NSCLC, melanoma, and urothelial bladder cancer. The incidence of atezolizumab-induced interstitial pneumonitis is less than 1%, with grades 3 and 4 accounting for 1.1-2.6% of cases, considered to be one of the lowest pneumonitis rates among ICIs. The most common CT changes suggestive of toxicity are the NSIP pattern and sarcoid reaction/lymphadenopathy.^56^

Durvalumab is a selective, human IgG1 monoclonal antibody that also blocks PD-L1. This drug is clinically active in urothelial carcinoma, hepatocellular carcinoma, head and neck squamous cell carcinoma, gastroesophageal cancer, and lung cancer.^57^ The overall incidence of DILD secondary to durvalumab is less than 5% but reaches 38% when the drug is combined with osimertinib, showing subclinical pulmonary infiltrates with diffuse GGO, bronchospasm, and bronchiectasis.^1^ ^,^ ^51^

### CTLA-4 inhibitors

Ipilimumab, a monoclonal antibody that blocks CTLA-4, is widely used in the treatment of melanoma, usually in combination with nivolumab (Figure 2C). The reported incidence of DILD is higher (10%) in cases of combined anti-PD1/PDL1 and anti-CTLA 4 treatment.^2^ Airway disease can occur, and COPD exacerbations following CTLA-4-based therapies have been reported, although such occurrences appear to be rare complications of ipilimumab use. Intrathoracic lymphadenopathy and a pattern suggestive of sarcoidosis are seen in 5-7% of patients treated with the combination of ipilimumab and nivolumab.^56^

### Conjugated antibodies

Trastuzumab-deruxtecan (T-DXd) is a novel antibody drug conjugate that consists of the anti-ERBB2 (HER2) monoclonal antibody trastuzumab and the topoisomerase I inhibitor deruxtecan. It is most commonly used in advanced breast cancer but also in the treatment of gastric and lung cancer. The most significant result of T-DXd-related toxicity is ILD, the incidence of which varies depending on the location of the tumor. A variety of radiological findings can be observed, such as OP, NSIP, hypersensitivity pneumonitis-like patterns, and ARDS. Up to 25% of patients with lung cancer treated with T-DXd develop pneumonitis. Because of the high toxicity rates, if the patient develops symptomatic ILD/pneumonitis (grade ≥ 2; Figure 2D), T-DXd treatment must be permanently discontinued, and corticosteroid treatment should be promptly initiated.^1^ ^,^ ^58^

## MISCELLANEOUS

Antiarrhythmics, antimicrobials, and immunosuppressants can also result in DILD, as described below. The pathogenesis is variable in these scenarios, including pulmonary drug deposition, hypersensitivity reactions, immune dysregulation, and endothelial injury.

### Mechanistic target of rapamycin inhibitors

**Figure 3:**
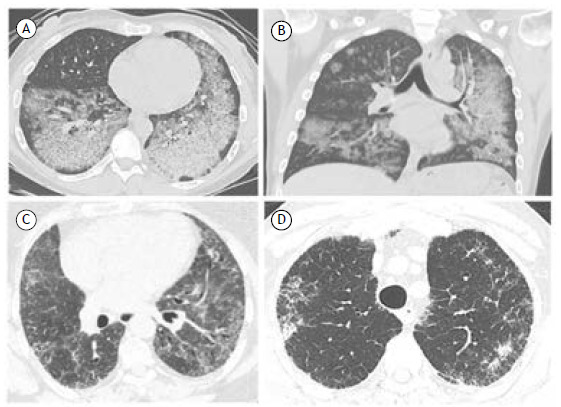
In A and B, chest CT showing ground-glass opacities and thickened interlobular septa, consistent with pulmonary alveolar proteinosis, in a patient using everolimus after kidney transplantation. In C, CT scan showing pulmonary ground-glass opacities, predominantly in the lower lobes, with traction bronchiolectasis in a 65-year-old female patient with RA after receiving methotrexate. In D, axial CT scan showing reticulated infiltrate and bilateral consolidations, predominantly in the upper lobes, in a male patient who received nitrofurantoin.

The mechanistic target of rapamycin inhibitors sirolimus and everolimus are potent immunosuppressive drugs used after organ transplantation and for the treatment of lymphangioleiomyomatosis. Estimates of the incidence of pneumonitis vary between 5% and 15%.^59^ These inhibitors are significant inducers of DILD, which manifests mainly as lymphocytic interstitial pneumonia, OP, or alveolar hemorrhage (Figures 3A and 3B).^2^ In patients with lymphangioleiomyomatosis, sirolimus therapy has been proven to be safe, and although pneumonitis has also been reported, it seems to be reversible and not to have an impact on long-term tolerability.^60^

### Methotrexate

Methotrexate is commonly used in RA, other rheumatic diseases, psoriasis, and some malignancies.^2^ Methotrexate-induced lung disease is the archetype of drug-induced pulmonary toxicity in patients with RA, usually occurring early in the course of therapy. Historically, methotrexate has been associated with RA-ILD; however, although it can induce subacute pneumonitis, a potentially lethal condition, its use does not seem to be associated with an increased risk of chronic fibrosing ILD; in fact, it might even be protective.^61^

The most common CT and histological findings of methotrexate-induced lung disease are similar to those of hypersensitivity pneumonitis. Other patterns include OP and DAD.^22^ The incidence of methotrexate-induced lung disease (Figure 3C) is estimated to be less than 1%.^62^

### Leflunomide

Leflunomide is a DMARD used in the treatment of RA that can lead to ILD exacerbation, accelerated formation of pulmonary rheumatoid nodules, and diffuse alveolar hemorrhage.^22^ Most patients present DILD within three months after starting leflunomide, with acute symptoms for a week or less. Bilateral GGO and DAD are the most common radiological and histopathological findings, respectively. Patients with preexisting ILD are particularly vulnerable to this complication, and leflunomide should therefore be used with caution in this population.^63^

### Amiodarone

Amiodarone is an antiarrhythmic widely used for supraventricular and ventricular arrhythmias, with a reported incidence of DILD of 1.2-8.8%^37^ and a mortality rate of 3-37%.^2^ ^,^ ^62^ A high cumulative dose is an important risk factor for amiodarone-related DILD. The combination of high doses (> 400 mg/day) and long-term use is more strongly associated with DILD than are dose or duration alone. The HRCT features include diffuse GGO, thickened interlobular septa, OP, ARDS, eosinophilic pneumonia, and diffuse alveolar hemorrhage. Lung nodules and masses may be seen, particularly in cases of subacute progression.^37^ ^,^ ^64^

**Figure 4:**
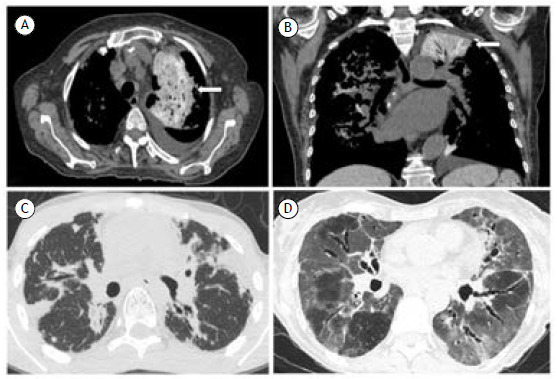
In A and B, axial and coronal CT scans, respectively, showing hyperattenuating lung parenchyma in the upper lobes (arrows) in a 77-year-old female patient with heart failure using amiodarone. In C, CT scan showing bilateral pleural thickening, predominantly in the upper lobes, in a male patient with lymphoma who underwent treatment with cyclophosphamide and developed pleuroparenchymal fibroelastosis. In D, CT scan showing diffuse pulmonary ground-glass opacities in a male patient after use of bleomycin for lymphoma treatment.

In patients receiving amiodarone, hyperattenuating lung parenchyma is commonly seen in areas of atelectasis on chest CT. This likely represents tissue deposition of iodine, analogous to what may be seen in the liver and thyroid,^37^ as depicted in Figures 4 A and 4B.

### Nitrofurantoin

Nitrofurantoin (a 5-nitrofuran derivative) is commonly used for the treatment and prophylaxis of urinary tract infections. In registry studies, DILD accounts for 16-48% of the nitrofurantoin-related adverse events reported.^64^ Nitrofurantoin-induced pulmonary toxicity occurs almost exclusively in women, mainly middle-aged or elderly women, because of their increased susceptibility to recurrent urinary tract infections and more frequent use of the drug. Acute presentations are more frequent, occurring within the first weeks of treatment, and may induce autoimmunity with ANA or antineutrophil cytoplasmic antibody positivity.^2^

Radiologically, nitrofurantoin-induced acute pulmonary toxicity manifests as diffuse GGO and consolidation with or without reticular changes, and traction bronchiectasis in patients with chronic disease. Histopathological findings may vary; acute disease is characterized by mild (and often eosinophilic) interstitial inflammation, whereas diffuse interstitial pneumonia with an NSIP pattern is predominant in chronic reactions,^2^ as illustrated in Figure 3D.

### Cyclophosphamide

Cyclophosphamide is an alkylating agent that is widely used in the treatment of autoimmune diseases and in hematological malignancies as part of a chemotherapy regimen.^65^ One potential late complication of treatment with alkylating agents is PPFE, which is characterized by fibrosis involving the pleura and subpleural lung parenchyma, predominantly in the upper lobes. The onset of PPFE after the administration of these treatments presents wide variability among the reported cases, ranging from 6 months to 16 years.^66^ Early onset pneumonitis and late onset pneumonitis with fibrosis (Figure 4C) have also been described as potential impacts of toxicity associated with the use of cyclophosphamide.^67^

### Bacillus Calmette-Guérin therapy

The intravesical or intrapelvic administration of bacillus Calmette-Guérin (BCG) is effective against urothelial cancer. Pneumonitis is a rare complication of this form of immunotherapy, seen in less than 0.7% of patients following the repeated administration of BCG. Micronodules with a miliary pattern, bilateral pulmonary opacities, and a reticulonodular pattern have been described. A hypersensitivity reaction rather than a disseminated BCG infection is suspected to be the pathogenesis of this disorder. Epithelioid noncaseating granulomas of the lung have been identified in several cases.^68^

### Sulfasalazine

Sulfasalazine is used worldwide for the treatment of ulcerative colitis and RA. The CT findings of DILD secondary to sulfasalazine use include GGO and consolidations with air bronchograms, consistent with OP, as well as interstitial opacities with pleural thickening in the upper lungs. Hypersensitivity pneumonitis occurs in some cases.^69^

## RADIATION RECALL PNEUMONITIS

Radiation recall is an inflammatory reaction within previously treated radiation fields precipitated by chemotherapy (taxanes, anthracyclines, alkylating agents, or pyrimidine analogs) or other medications (tamoxifen, simvastatin, levofloxacin, or isoniazid) and can occur in several different systems.^70^ The precise pathophysiological mechanism of radiation recall pneumonitis remains unclear. One hypothesis is that radiotherapy sensitizes immune cells and local vasculature, resulting in greater toxicity in previously irradiated areas than in non-irradiated areas after exposure to certain agents.

The classical radiologic manifestations of radiation recall pneumonitis include GGO, diffuse opacities, and patchy consolidation, which corresponds to the shape and size of the radiotherapy field. Radiation pneumonitis commonly occurs in patients treated with radiotherapy to the lung. Radiation recall pneumonitis may occur in the previously irradiated lungs of patients after the administration of inciting agents.^71^ Histological features include interstitial edema, hemorrhage, and a fibrinous exudate in the early stages of the disease with later distortion and fibrosis.^72^ However, a lung biopsy is very rarely needed to establish the diagnosis of radiation recall pneumonitis.

## DIAGNOSIS

The symptoms of DILD are nonspecific, and clinicians should beware of late respiratory symptoms in patients treated with any drug that may cause pulmonary toxicity. When there is a possibility of DILD, the website *www.pneumotox.com* may be useful to check potential toxicities of the drug involved. Disease onset varies from days to even years and is usually unpredictable. Symptoms of DILD include dyspnea, cough, and fever, evolving to respiratory failure with hypoxemia in some cases.^2^ Chest CT has high sensitivity for detecting ILD features and is the imaging modality of choice. However, there is no specific pattern for DILD, given that the various forms of interstitial involvement are commonly seen in other ILDs.^62^

The diagnosis of DILD is therefore based on the exclusion of other causes, including infections, heart failure, lymphangitic carcinomatosis, connective tissue disease (e.g., RA or systemic sclerosis), and inflammatory bowel disease. Bronchoscopy is useful for investigating differential diagnosis, such as infections or malignancies, and BAL may show lymphocytosis, eosinophilia, or alveolar hemorrhage, thus usually precluding the need for lung biopsy.^56^ Improvement in symptoms following discontinuation of the offending drug favors the diagnosis of DILD, as does recurrence after reexposure.

## TREATMENT

Discontinuation of the culprit drug is the mainstay of treatment. Few studies have evaluated the treatment of DILD, and the current guidelines, which are based on observational reports and clinical experience, have not been standardized or validated in prospective clinical trials.^2^ ^,^ ^56^ Pharmacological treatment of DILD is currently based on systemic steroids, and the dosing varies according to severity and the CT pattern.

**Figure 5:**
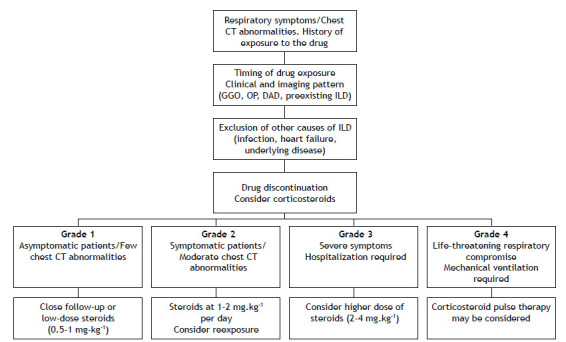
Management of patients with drug-induced lung disease. GGO: ground glass opacities; OP: organizing pneumonia; DAD: diffuse alveolar damage; ILD: interstitial lung disease.

Severity grades are well-established guidance in the management of toxicity by ICIs (Chart 1). In grade 1 pneumonitis (asymptomatic with only radiographic changes), close follow-up or treatment with low-dose steroids (0.5-1.0 mg·kg^−1^) can be employed, with drug maintenance (Figure 5). For grade 2 pneumonitis (symptomatic with CT changes), treatment with steroids at 1-2 mg·kg^−1^ per day seems to be adequate and reexposure to the drug can be considered. For grade 3 or higher pneumonitis (severe symptoms and limiting self-care activities of daily living requiring supplemental oxygen), a higher dose of steroids (2-4 mg·kg^−^1) is recommended, together with drug withholding. Corticosteroid pulse therapy may also be considered in severe cases. Steroid tapering should be conducted very slowly and carefully over the course of at least 6 weeks, given that relapses of pneumonitis have been reported during the weaning period. Persistent changes on chest CT can be useful to guide steroid tapering over time.^6^ ^,^ ^56^ ^,^ ^73^

Although the use of immunosuppressive drugs for DILD, such as infliximab, mycophenolate mofetil, and cyclophosphamide, is not completely established, treatment with such drugs may be considered in severe and refractory cases, or when steroids are needed for long periods.

## FINAL CONSIDERATIONS

Pulmonary drug toxicity is an adverse event that is common and relevant. The incidence of such toxicity is rising because of the increasing number of new medications included in the list of drugs that can cause DILD, especially in the treatment of autoimmune diseases and cancer. Given the large number of drugs that potentially cause pulmonary toxicity, various patterns of DILD on CT have been described and the prognosis is highly variable. The diagnosis of DILD requires the exclusion of alternative causes and can be a challenge, especially in patients with preexisting ILDs and using several drugs concomitantly. However, lung biopsy is rarely needed in order to confirm the diagnosis. Discontinuation of the offending drug is essential in the treatment, and corticosteroids are frequently used in acute conditions. The decision on whether to rechallenge a patient with the same drug after a prior episode of drug-induced pulmonary toxicity should be made on a case-by-case basis, preferably in the setting of a multidisciplinary discussion. 
